# The inseparable relation between foresight and uncertainty

**DOI:** 10.1093/eurpub/ckac129.120

**Published:** 2022-10-25

**Authors:** H Hilderink, D Moye Holz

**Affiliations:** RIVM, Bilthoven, Netherlands

## Abstract

This presentation starts with an interactive mentimeter poll to involve the audience. They will be asked several questions about what they find important in dealing with uncertainty in public health foresight studies.

**Background:**

The future is per definition uncertain. Our knowledge about the future is obviously limited, especially when we look further into the future. Next to limited knowledge, people also have different ideas about what they consider a desirable future. All these aspects of uncertainty play a role when doing a foresight study. One of the challenges when doing a foresight study is to address these uncertainties in a systematic manner.

**Methods:**

The cognitive uncertainty addresses the lack of knowledge, which can easily be extended to the information that can be inaccurate or unreliable. These cognitive uncertainties can vary widely, and can be classified according to location, level and nature of the uncertainty. Especially in foresight studies with a strong quantitative character, the cognitive uncertainties can be crucial and can form the basis of developing scenarios. Next to the cognitive uncertainties, normative uncertainties can be distinguished which address desirable futures. These represent the obvious differences in norms and values that people have when valuing health. There are several examples of public health foresight studies that include a proper consideration of uncertainties involved.

**Conclusions:**

Applications of foresight studies in the field of public health are still limited. However, the recent years we see a broadening of initiatives of doing a foresight study, including addressing uncertainty in an more systematic way. Especially in public health, the normative uncertainty might play a rather relevant role.

